# A frequency-based linguistic approach to protein decoding and design: Simple concepts, diverse applications, and the SCS Package

**DOI:** 10.5936/csbj.201302010

**Published:** 2013-03-29

**Authors:** Kenta Motomura, Morikazu Nakamura, Joji M. Otaki

**Affiliations:** a The BCPH Unit of Molecular Physiology, Department of Chemistry, Biology and Marine Science, University of the Ryukyus, Senbaru, Nishihara, Okinawa 903-0213, Japan; b Department of Information Science, University of the Ryukyus, Senbaru, Nishihara, Okinawa 903-0213, Japan

## Abstract

Protein structure and function information is coded in amino acid sequences. However, the relationship between primary sequences and three-dimensional structures and functions remains enigmatic. Our approach to this fundamental biochemistry problem is based on the frequencies of short constituent sequences (SCSs) or words. A protein amino acid sequence is considered analogous to an English sentence, where SCSs are equivalent to words. Availability scores, which are defined as real SCS frequencies in the non-redundant amino acid database relative to their probabilistically expected frequencies, demonstrate the biological usage bias of SCSs. As a result, this frequency-based linguistic approach is expected to have diverse applications, such as secondary structure specifications by structure-specific SCSs and immunological adjuvants with rare or non-existent SCSs. Linguistic similarities (e.g., wide ranges of scale-free distributions) and dissimilarities (e.g., behaviors of low-rank samples) between proteins and the natural English language have been revealed in the rank-frequency relationships of SCSs or words. We have developed a web server, the SCS Package, which contains five applications for analyzing protein sequences based on the linguistic concept. These tools have the potential to assist researchers in deciphering structurally and functionally important protein sites, species-specific sequences, and functional relationships between SCSs. The SCS Package also provides researchers with a tool to construct amino acid sequences *de novo* based on the idiomatic usage of SCSs.

## Introduction

In the mid-20th century, molecular biology revolutionized biological sciences through the discovery of the molecular information flow through which proteins are built based on DNA sequences. This discovery has been known as the central dogma of molecular biology [1]. Since its discovery, the “secret of life” or the “essence of life”, in a popular sense, has often been attributed to DNA or genes. The molecular function of DNA is quite simple and static and can be summarized as follows: DNA stores and supplies information regarding how proteins are built. Proteins perform almost all the functions necessary for life, including the retrieval and maintenance of genetic information. In this sense, the secret or essence of life likely resides not only in DNA but also in proteins.

The language of DNA (the information coded by DNA) simply involves the use of triplet codons to specify protein amino acid sequences. These amino acid sequences, in turn, specify their own protein structures and functions. This intra-molecular information decoding process, i.e., folding process, is the final step that completes the biological information flow delineated in the central dogma. How does this intra-molecular information flow take place? Anfinsen partially answered this question by stipulating the general rule known as Anfinsen's dogma [2]. Anfinsen's dogma states that structural and functional information about a protein is coded in that protein's amino acid sequences and nowhere else. This leads researchers to question how the structural and functional information is coded within amino acid sequences. Unfortunately, Anfinsen's dogma does not provide any clues in this regard.

Since early endeavors to decipher protein structures [3, 4], many protein three-dimensional structures (as of October 16, 2012, 85,435 structures in the Protein Data Bank [5]) have been determined with atomic or subatomic resolution, greatly improving our understanding of how these proteins work. BLAST [6], the most influential alignment tool, and other computational tools have also greatly improved our understanding of primary structures. As a cumulative result of these and other multi-faceted protein studies, there have been many attempts to rationally design proteins [7–10]. Nonetheless, no reasonable answer upon which most scientists can agree has been found for this half-century-old problem regarding the intra-molecular information codes contained within proteins [11, 12]. Today, we remain far from a complete understanding of the protein codes of amino acid sequences.

A brave new idea may be required to break the protein codes written in amino acid sequences [13–15]. Strictly speaking, our idea of analyzing amino acid sequences based on frequencies (also called composition, occurrence, and count) of amino acids and short constituent sequences (SCSs) is neither new nor brave [15, 16]. However, thanks to advancements in computer technology and the accumulation of sequence and structure records in databases, this classical idea can be realized without using a supercomputer. We are now able to perform exhaustive frequency searches for all possible *n*-aa SCSs (or words) when *n* is reasonably small. This paper reviews the current status of the frequency-based approach, focusing on our simple linguistic approach, while, for simplicity, excluding other related but more complicated approaches [e.g., 16-19]. We conclude with possible future directions aimed toward cracking protein codes. More philosophical and basic discussions can be found in a previous review article [15].

## Beyond alignment-based analysis

There is no doubt that alignment-based programs are very powerful tools for examining relationships among proteins with regard to amino acid sequences. However, protein biochemists know that different sequences can result in similar three-dimensional structures and functions and that identical sequences can have different structures and functions in a context-dependent manner [20], suggesting that information extraction by alignment-based methods is not sufficient to understand the folding process. For example, despite the three-dimensional structural similarities in G-protein-coupled receptors (GPCRs), analyses and classification of GPCRs cannot rely entirely on simple alignment methods because of the lack of significant sequence similarities except particular short constituent sequences (SCSs) in restricted sites. This is one of the most important reasons for the development of alignment-free methods for sequence comparison. Many of the alignment-free methods use tuple or n-gram analysis [16–19], principal component analysis [21], and other advanced computational methods. Our group obtained reasonable results for GPCR analyses in an alignment-free fashion using membrane topology [22] and self-organizing map (SOM) [23]. However, these methods are more or less specific to GPCRs, and most alignment-free algorithms, including our self-organizing map [23], are often mathematically complicated. We looked for a simple operation and endeavored to decode proteins using a more general approach with the hope of increasing applicability to all proteins.

Alignment-based programs also suffer some fundamental limitations. They are not particularly successful at handling gaps or length differences generated by recombination and shuffling. Furthermore, high level comparisons between species (such as comparisons at the genomic level) are difficult to obtain by alignment alone. Although short similar sequences can be used as seeds for homology searches [6], short similar sequences are generally considered to be biologically insignificant noise events that occur with particular probabilities. However, specific short sequences could be functionally positioned even if their probability of being located at that particular position is close to the background noise levels of the alignment search programs.

To illustrate these limitations, we use the conceptual analogy between a protein and an English sentence, both of which are constructed by short constituent sequences (SCSs) or words, respectively. Consider the following two sentences:

I usually ate an apple when I was a boy because I liked it.

Among fruits, apple was my favorite in my childhood days.

These two sentences do not have any similarity at all except “apple” and “was”. Nevertheless, they have almost the same meanings. In these sentences, “apple” is not noise but the key word that implies the similar meanings of these two sentences. Of course, we can also imagine a very different sentence, for instance, the following:

I did not like apples at all when I was a little boy.

Except for the word “apple(s)”, this sentence (3) has no similarities with sentences (1) and (2). The meanings of sentence (3) and sentences (1) and (2) are also very different. Nonetheless, the word “apple(s)” still provides common ground because apple(s) is the key word and has the same meaning in all three sentences. Moreover, despite its opposite meaning, the topic of sentence (3) is similar to that of sentences (1) and (2). In analogy with proteins, sentences (1) and (2) may share identical binding sites and ligands. Though they have very different amino acid sequences, their folded structures may be similar. Likewise, though sentence (3) may be more structurally and functionally different, it may still have some functional similarity to sentences (2) and (3) at a sub-molecular level.

Furthermore, one can change the key word, apple(s), in one or more of these sentences to a different fruit, for instance, orange. In this case, sentence (4) will read:

I did not like oranges at all when I was a little boy.

Sentences (3) and (4) are almost identical except for their key words, apple or orange. In the same way, ligand specificity may be very different because of a difference in binding sites (apple vs. orange). They have high alignment scores and are considered to be similar proteins, but it is important to note that the non-aligned words, apple and orange, are as important as the aligned words. If one examines a sufficiently large number of sentences, it will be discovered that “apple” and “orange” emerge in the context of a group of other words such as “like”, “fruit”, and “eat”. And it is possible to calculate how often these words appear in the vicinity of one another.

The discussion above assumes that the units of amino acid sequences and English sentences are SCSs and words, respectively. This standpoint appears to be justifiable considering the structural and functional importance of such small protein sequences [24, 25]. In the early stages of biological evolution on Earth, small peptides were likely more extensive than complex proteins in terms of functionality. It is suggested that the earliest protein had the size of 7 amino acids [26]. Given that a small peptide contains just a few “words”, the SCS may constitute an evolutionary unit from an early stage of biological and chemical evolution. The SCS may also be a unit of structure as well as function. We acknowledge that the entire protein structure is also an important component of the protein's code. In this regard, artificial intelligence systems, which can identify hidden patterns that are not recognized readily by human intelligence, may be useful [e.g., 23].

## Simple frequency-based approach

Overcoming alignment-associated limitations could eventually lead researchers to an understanding of sequence-structure relationships. We have focused on the frequency-based analysis of SCSs. The technical advantage of this SCS analysis is that SCSs in a given sequence are “compared” to the entire non-redundant amino acid (nr-aa) database simultaneously and without any sequence-imposed restrictions [13–15]. We use the term “SCS” in this paper, although other groups have used “oligopeptides”, “oligomers”, and others. It is important to note that, because there are only 20 amino acids, SCS repertories are limited in number. Theoretically, there are exactly 8,000 triplets (3-aa SCSs, trimers, or tripeptides) that constitute all possible combinations of the 20 amino acids (20^3^). Similarly, there are theoretically 160,000 quartets (4-aa SCSs, tetramers, or tertapaptides) and 3,200,000 pentats (5-aa SCSs, pentamers, or pentapeptides). The post-genome era has produced large protein databases that are readily available to researchers and that are usable in comparison with the large number of SCSs.

Our simple strategy is to count the number of each SCS species in a large database. Count value (occurrence or frequency), which we designate *R* for real count, is assigned to each SCS species when a database is defined. On the other hand, we can simply calculate the probabilistic occurrence of a given SCS species in the database based on amino acid occurrence. This expected count value, which we designate *E*, can be calculated easily by multiplying occurrences of constituent amino acids. In the case of a triplet, *E* is calculated as follows:

$$E=Q•P_{1}P_{2}P_{3}$$

where *Q* is the total number of existing triplets in a database, and *P*_*1*_, *P*_*2*_ and *P*_*3*_ (derived from the occurrence(s) of each amino acid in that database) are the probabilities that each amino acid appears at a given position. The probabilistically estimated count *E* does not consider influences from nearby amino acids and thus cannot be used singularly as a frequency indicator for real proteins. We are interested in the differences between the real and expected counts (of each SCS). The differences are summarized via the availability score, which is simply expressed as follows:

A=(R-E)/E=(R/E)-1

In this equation, *A*, *R*, and *E* indicate availability score, real count, and expected count, respectively. Availability scores are assigned to all SCS species when a database is defined. In the case of the triplet, we have a list of *R*, *E*, and *A* for all 8,000 SCSs that are associated with a particular protein database. We primarily use the nr-aa, which is considered the universal proteome.

Availability scores are usually not zero. For some SCSs, the availability scores are very large, while, for others, they are –1 (non-existent) [14]. The availability score pertains to an “unexpected” bias on SCS usage that cannot be explained from the expected usage of amino acids. The origin of this bias is not entirely apparent, but it is likely to be evolutionary [14]. One study states that the codon number is related to this bias [27].

Fortunately, our findings regarding SCS usage have been confirmed independently by other groups [28, 29]. We now know that there are rare or non-existent SCSs in the nr-aa database (i.e., the universal proteome), and they can be synthesized chemically and biologically with little difficulty [14]. Non-existent SCS peptides have been analyzed, and, when contained in the sequence, have been suggested to disrupt a folding process [30].

## Optimal SCS length

We used 3-aa, 4-aa, and 5-aa SCSs in our research including the SCS Package (see below). But what is the optimal SCS length? Rare or non-existent SCSs in a given database of interest (such as a secondary structure database) can be found relatively easily if a set of 5-aa SCSs is used [14, 15, 31, 32]. This is because the repertoire of 5-aa SCSs (20^5^ = 3.2 × 10^6^) is large enough to describe the sequence complexity of proteins and small enough to find similarities among different proteins.

Practically, longer SCSs may not be very useful. It should be noted that repertoire of *n*-aa SCS (i.e., all possible combinations of *n* amino acids) dramatically increases as SCS length (*n*) increases [14, 15]. For example, the repertoire of 6-aa SCSs (20^6^ = 6.4 × 10^7^) is already comparable to the number of SCS samples in the nr-aa database [14]. As a result, many of theoretically possible 6-aa SCSs or longer SCSs do not occur at all in the nr-aa database. This situation makes 6-aa or longer SCSs unsuitable for analyzing sequence complexity of proteins.

Therefore, we state that an optimal SCS length is 5 amino acids. Interestingly, this is in concert with other independent analysis called structural alphabet, where 16 representative “proteins blocks” (5-aa structural fragments) define three-dimensional structures [33, 34]. However, we think that there is no need to exclusively focus on a particular SCS length. We believe that 3-aa, 4-aa, and 5-aa SCSs are all unique, and one of them (or all of them) can be used on a case-by-case basis. For example, in our availability plot program (see below), all three types of SCSs were used, but in our idiom search programs (see below), we concentrated on 3-aa SCSs, just for simplicity.

## Word-oriented applications: from structural predictions to vaccines

Our frequency-based approach has a high potential for various applications. First of all, it can be used as a tool to examine amino acid sequences in one dimension. This was realized as “availability plot” (see below). Simply because the frequency-based approach is entirely free from alignment, it may be productive to efficiently combine both approaches to observe both sides of proteins. The frequency-based approach could improve alignment algorithm when two very different sequences show similar structure and function.

Additionally, secondary structure characterization is one of the important applications of the frequency-based word analysis [18, 19, 31, 32, 35]. Through the construction and analysis of secondary-structure-specific databases, we have shown that some SCSs are favored in α-helices and others in β-strands [31]. These structure-specific SCSs may be used as markers or discriminant sequences for particular secondary structures. Similarly, we have demonstrated that parallel and antiparallel β-strands differ in their amino acid compositions and the availabilities of their SCSs [32]. Although these results have been expected historically, this is the first time that they have been demonstrated conclusively. Cap structures of helixes and sheets [36] may be analyzed similarly and thus identify the beginning and end of a given secondary structure. C-terminal sequences have already been analyzed with success [37]. These studies are the first step to decoding amino acid sequences in order to understand the sequence-structure relationships. Using similar methodologies, we can search for SCS signatures in any database system. For example, phylum-dependent signatures have been reported [38]. This example demonstrates the power of frequency-based analysis when proper and reasonably large databases are constructed. We have also performed a similar study to examine species relationships using species-specific databases [39].

Non-existent SCSs may also have important applications. The first application of a “wet” system has just been published [40] and shows that rare or non-existent SCSs are useful as immunological adjuvents. The idea of using peptides in vaccines is not new [24, 25], but using non-existent SCSs in vaccines is a completely new idea. We have to recognize that the number of combinatorial sets of amino acids is almost infinite [15]. Proteins on the earth constitute a very tiny, possibly negligible, fraction of the entire protein space. The possible use of non-earth-type protein space provides us with a tremendous opportunity to explore artificial proteins [39].

These dry and wet applications will continue to emerge with focuses on specific SCSs. As such, we refer to them as word-oriented applications in contrast to context-oriented applications, which are discussed below.

## Context-oriented applications: from words to sentences

The applications discussed thus far pertain to identifying specific SCSs to infer secondary structures, or using SCSs in vaccines. These word-oriented applications are a direct extension of compositional analyses of amino acids. On the other hand, just as words are connected in sentences, SCSs are connected in the entire amino acid sequences of proteins. Although they cannot be completely differentiated from word-oriented applications, we refer to analyses of proteins in terms of their entire amino-acid (sentence) structure as context-oriented applications.

There is a rank-frequency relationship in quantitative linguistics known as Zipf's law [41, 42]. To illustrate how the rank-frequency relationship is examined, let us consider the following sentences from one of our recent papers [43].

The amino acid sequences of proteins determine their three-dimensional structures and functions. However, how sequence information is related to structures and functions is still enigmatic. In this study, we show that at least a part of the sequence information can be extracted by treating amino acid sequences of proteins as a collection of English words, based on a working hypothesis that amino acid sequences of proteins are composed of short constituent amino acid sequences (SCSs) or ‘‘words’’. We first confirmed that the English language highly likely follows Zipf's law, a special case of power law.

The frequency of words (or the number of words, or word count) in the above sentences can be summarized as follows (words with only one count are not listed): the (3 times), amino (4), acid (4), sequences (6), of (7), proteins (3), structures (2), and (2), functions (2), information (2), is (2), we (2), that (3), a (4), English (2), and words (2). Now, based on the frequency data, these words are ranked in descending order as follows: Rank 1, of (7 times); Rank 2, sequences (6); Rank 3 amino (4), acid (4), and so on. This way, a given word is associated with two numerals, rank and frequency. One can now make a rank-frequency plot. The mathematical relationship between rank and frequency is known as Zipf's law, or more generally, power law.

Zipf's law states that, in a natural language system, the occurrence or frequency of words is inversely proportional to their ranks. Importantly, this relationship is valid at least over a few orders of magnitude. The scale-free nature of these rank-frequency relationships has been thought to originate from communication tradeoffs (between the speaker and the hearer) described by the least effort principle [42, 44]. Speakers try to minimize their verbal efforts to convey their ideas, whereas hearers try to minimize the process of understanding. Both prefer brief expressions, but speakers can use ambiguous words at will, whereas hearers prefer unambiguous words that better enable them to understand expressions instantly and without much effort.

The basis of natural language evolution discussed above may have conceptual similarities to the relationships between primary and tertiary structures. The primary structure changes randomly via random mutation during evolution. As such, this process has no constraints and produces functionally ambiguous changes. However, the tertiary structure has functional constraints, and unambiguous functional changes are preferred. In light of the above analogy, we have compared protein amino acid sequences and English sentences in a manner inspired by Zipf's law, or more generally, power law [43].

Our recent study has demonstrated a scale-free nature of protein amino acid sequences that is comparable to or even larger than that of the English language [43]. However, dissimilarity is also apparent. A unique feature of proteins is the sharp deviation of their low frequency SCSs from a straight line in rank-frequency plots. We also observed species-specific trends in SCS distribution patterns. Further research may reveal a natural language with a similar distribution pattern to that of proteins. Likewise, we may be able to find a specific proteome that has a similar distribution pattern to that of the English language.

Encouraged by this analogy, we devised a so-called availability plot as a tool for finding possible functional sites [43]. This availability plot is implemented as part of the SCS Package (see below). High availability sites correspond to known motifs in some but not all proteins [43]. The fact that there are high-availability sites that do not correspond to known motifs may simply mean that we have not discovered new motifs at those or other functionally important sites. Alternatively, high-availability sites may have no apparent biological significance. We also must examine low availability sites, which may serve as key SCSs in particular sequences.

A related study proposed “protein conservation profile” [45]. This is a simple frequency plot along a given amino acid sequence and thus different from our availability plot, which shows availability scores of SCSs along a given amino acid sequence. Nonetheless, their claim that the frequency plot can reveal a common feature of prokaryotic proteins [45] is supportive of our frequency-based approach to protein decoding.

Again, using linguistic ideas to analyze proteins is not new. In fact, numerous studies have used linguistically oriented algorithms for protein analysis, and at least one study has compared protein sequences to human languages [46]. However, our recent study [43] is thought-provoking in that the rank-frequency plots therein reveal both similarities and dissimilarities between protein sequences and the English language. These comparisons between proteins and English may pave the way for yet a new method of analyzing protein sequences, in addition to availability plots (see below).

## The SCS Package

**Figure 1:**
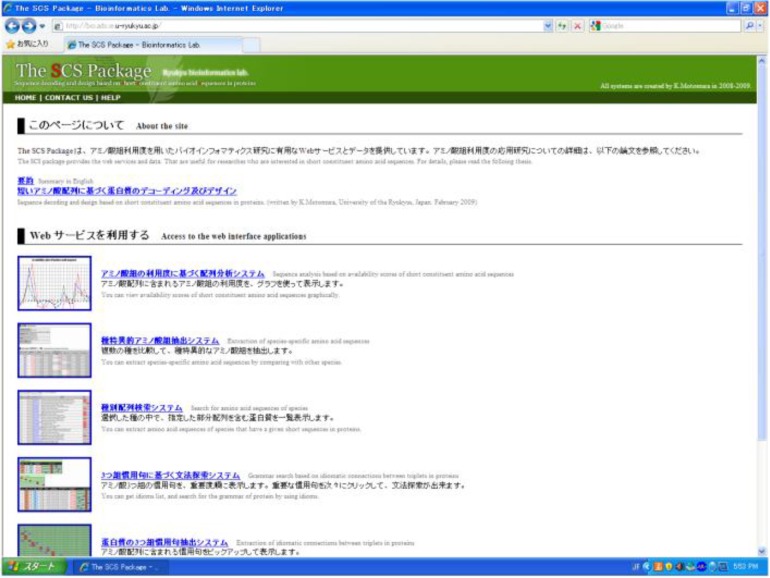
**Home page of the SCS Package**. Five web-interactive applications are listed, and these are freely accessible at http://bio.ads.ie.u-ryukyu.ac.jp

To help researchers get accustomed to the simple concept of availability and to examine proteins or proteomes of interest based on SCS availability scores, we have developed a collection of web-based applications called the SCS Package (Figure 1), which is freely accessible at http://bio.ads.ie.u-ryukyu.ac.jp. This web server is primarily in Japanese but English is also used. For the most part, the applications are very easy to use, such that the programs may be run easily without requiring the reading of explanatory sentences. It is important to recognize that the SCS Package is not built to handle collections of data automatically. Users are expected to use this web service manually. The SCS Package contains five different applications, the icons for which are located on the home page of the SCS Package URL site. Below, we delineate how to use the applications from the top down (in the order shown on the home page). The SCS Package contains the pre-calculated on-board SCS databases that were produced from the nr-aa database and English Wikipedia [39, 43]. The databases upon which the availability plot is based were updated on August 2012. Other programs are based on the databases downloaded on November 2009. Development of the SCS Package and other related programs and their preliminary applications were presented in the thesis of one of the authors (written in Japanese but containing an English summary) [39], which is also available via the SCS Package site.

### Availability plot program

**Figure 2:**
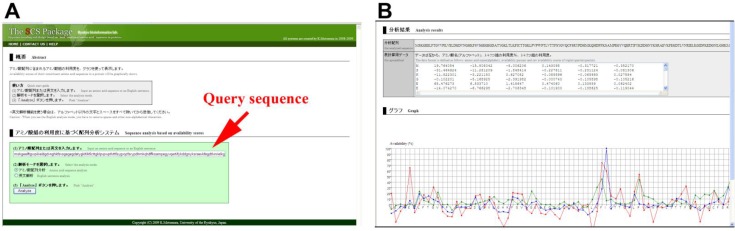
**Availability plot program**. (**A**) Input page of the first program of the SCS Package. User puts the query sequence in the box provided. In this case, it is green fluorescent protein (GFP) from *Aequorea victoria* (GenBank Accession No. AAA27722). (**B**) Output page. Excel-friendly numerical outputs (top) and graphical outputs (bottom) are shown. The *X*-axis is protein amino acid sequence, and the *Y*-axis is relative availability (%). To calculate the relative availability, the highest peak of the original availability score in this sequence was set at 100%, and other scores were proportionally adjusted. Graphs of 3-aa, 4-aa, and 5-aa SCSs (triplet, quartet, and pentat) are shown in red, blue, and green, respectively.

The first program pertains to the availability plots discussed above (Figure 2A). This application analyzes distributions of availability scores of 3-aa, 4-aa, and 5-aa SCSs throughout a given amino acid sequence. The resulting availability plot is useful in examining high and low availability sites. In a fashion similar to the well known Kyte-Doolittle hydropathy plot [47], availability scores are assigned to all SCSs and are connected by lines (Figure 2B). Availability plots of triplets (3-aa SCSs), quartets (4-aa SCSs), and pentats (5-aa SCSs) are shown in red, blue, and green, respectively, in the identical graphic window. The residues at the end of protein chains do not have availability scores, because they do not form these SCSs. In the graphics, the *X*-axis is the query amino acid sequence, and the *Y*-axis corresponds to the relative availability scores that are calculated by setting the highest raw availability score in the query sequence to 100% and adjusting the other scores proportionally. We encourage users to make their own graphs using the spreadsheet-friendly output data and Microsoft Excel. The plot results may be compared via motif analysis, hydropathy plots, and other methods, in order to infer the functionality of specific sites within that particular sequence. We have demonstrated that availability plots can identify known motifs in at least some proteins [43].

### Species specificity search programs

**Figure 3:**
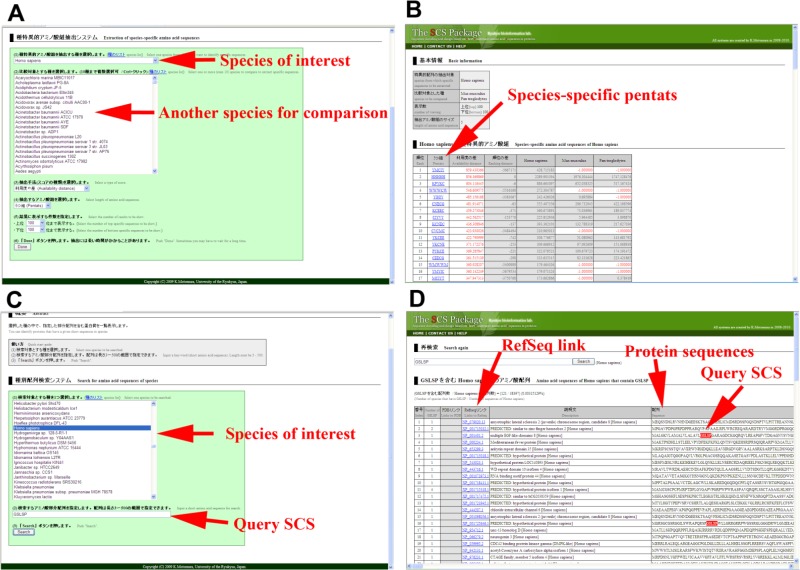
**Identification of species-specific amino acid sequences**. (**A**) Input page of the second program of the SCS Package. The user first specifies a species of interest, and then specifies another species for comparison (up to 10 species simultaneously). The user can choose either availability distance or rank distance and specifies the number of amino acids in the SCSs to be analyzed. The user further specifies how many top and bottom specific sequences will be shown. (**B**) Output page. Identified species-specific SCSs are listed. One can click on the SCSs shown in blue and examine the list of proteins containing each SCS. The availability scores of three species, in this case *Homo sapiens*, *Mus musculus*, and *Pan troglodytes*, are shown on the right side of this output table. (**C**) Input page of the third program of the SCS Package. The user specifies a species of interest and query SCS. (**D**) Output page. Proteins that contain the query SCS in a species of interest are listed with RefSeq links.

The second application extracts species-specific SCSs between two species or among several different species (Figure 3A). This program takes full advantage of the alignment-free nature of the availability-based analysis. In comparing two or more sequences, there is no need for any sequence similarity among them. Closely related species, such as human and chimpanzee, often show almost identical sequences. Since almost all proteins in a species have orthologous and paralogous proteins in another related species, it is difficult to discern species-specific features in a given proteome. SCS distribution patterns may help solve this problem. For example, a user can specify *Homo sapiens* as a species of interest and then select one (or up to 10) species from the list to be compared with *H. sapiens*. Users can choose the type of score, either availability distance or ranking distance, to be compared, noting that ‘distance’ means simple subtraction. Users can also choose lengths of SCSs from 3 to 5. When a user specifies one species, say *Pan troglodytes*, to be compared with *H. sapiens*, the availability distance or rank distance is calculated, and the top 100 and bottom 100 (specified by the user) SCSs are listed on the output page (Figure 3B). When a user specifies two species to be compared to *H. sapiens*, the availability score of *H. sapiens* is doubled (due to two species), and the availability scores of both species are subtracted, giving the availability distance.

The third application is no more than a simple search program for given SCSs in a given species (Figure 3C). A user specifies a single species and inputs an SCS of interest. The program then shows a list of proteins that contain the specified SCS in the specified species. For example, when a user specifies *H. sapiens* and KENTA as the SCS of interest, the program shows a list of proteins that contain KENTA (Figure 3D).

### Idiom search programs

**Figure 4:**
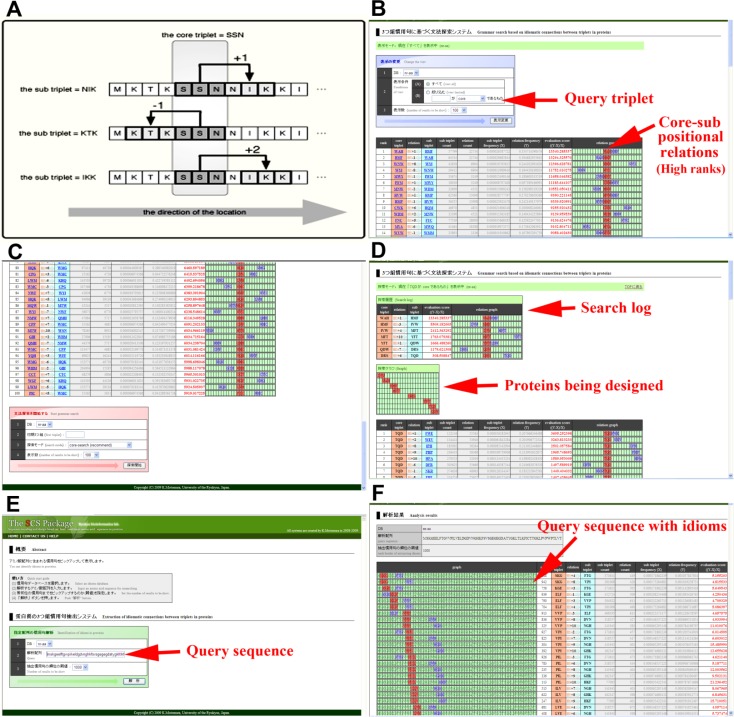
**Grammar search based on idiomatic connections between triplets in proteins**. (**A**) Definition of positional relations between core and sub triplets. (**B, C**) Input page of the fourth program of the SCS Package. As a default, this page shows a list of idioms that have high evaluation scores. Positional relations between the core and sub triplets are shown on the right side of the output table. (**D**) Interactive output page. Using idiomatic connections between two triplets, the user can design a protein amino acid sequence using a single core triplet as a seed. (**E**) Input page of the fifth program of the SCS Package. The user puts a query sequence in the box provided. In this case, the GFP sequence was used, as in Figure 1. (**F**) Output page. The query sequence is shown on the left side of the output table, in which core triplets are shown in red and sub triplets in purple. The core triplet located in the vicinity of the N terminus is shown at the top of this list. Idiomatic connections are found throughout the GFP sequence, which most likely signifies a β-barrel structure. In other proteins, idiomatic connections are not so abundant. Thus, such idiomatic sites may be functionally important in proteins. This application may be used together with the availability plot program.

The fourth program is a “grammar search” based on idiomatic connections between triplets in proteins. Some definitions are necessary here (Figure 4A, B). “Core triplet” refers to a triplet of interest, and “sub triple” refers to a triplet that is strongly associated with the core triplet. “Relation” means a positional relation between the core and sub triplets. For example, relation +1 means that a sub triplet is just next to the core triplet on the right (i.e., on the C-terminal side). The “sub triplet count” is the raw number of sub triplets found anywhere in the nr-aa database. This sub triplet count is divided by the total number of triplets in the nr-aa database, 2,173,898,133, producing the sub triplet frequency (*X*). This is a probability that this triplet is found at a given position in this database. Additionally, “relation count” indicates the number of a given idiom (core and sub triplets at the particular positions just as its relation indicates). This relation count is divided by the total number of potential idioms that have the same core triplet but have any sub triplet at the position indicated, producing the relation frequency (*Y*). In this grammar search program, the denominators for producing *Y* values are not indicated explicitly, but one can calculate them, if necessary, by the numerators and *Y* values shown. The relation frequency *Y* indicates a probability of having a particular idiom, but a *Y* value may be large simply because its triplet is abundant in the database. This is why the evaluation score is defined as (*Y* – *X*) / *X*. A high evaluation score means that connections between the core and sub triplets at the given positions (i.e., idioms) are frequently observed in comparison with a frequency of that sub triplet without positional restrictions.

One can use the above program to identify frequently used triplets within a ±10 amino acid range from a given triplet in question (Figure 4B, C). Opening the first page of this program reveals a top 100 list of triplets having strong idiomatic connections with other triplets. This is the default screen. Users can let the program show up to the top 1,000 idioms (Figure 4B). Furthermore, one can specify a triplet and let the program automatically show a list of idioms of high ranks (from rank 1 to 1,000) that contain the specified triplet as a core or sub triplet.

It is possible to perform sequential searches with this program using the search log and relation graph (Figure 4C, D). One can click on a core triplet and then a sub triplet in the list and perform several sequential idiom searches to obtain a high-frequency cluster of a long stretch of amino acids. Alternatively, one can start a sequential idiom search by specifying the triplet of interest in the query box at the bottom of this page (Figure 4C). The resultant amino acid stretches (Figure 4D) may serve as clusters of idioms that show ideal relations among triplets, and they may correspond to real protein sequences. In this way, ideal artificial protein sequences can be devised. We believe that this program provides a prototype with which a future protein designer can “write” protein sentences using a computer.

It is possible to design non-existent proteins in a similar fashion to that described above, but the algorithms are more complicated and are not built into the SCS Package. Nonetheless, we have successfully designed long, artificial, non-earth-type proteins [39], which will be formally published elsewhere. Because non-existent protein space is so vast, such non-existent (or non-earth-type) artificial proteins will open up a whole new field of protein engineering.

The fifth and final application identifies spatial relationships between SCSs in a given amino acid sequence (Figure 4E). Rather than examining particular triplets and their associated triplets via the program discussed above, researchers are often interested in a particular protein. Using the fifth application, one can enter the entire sequence of a particular protein of interest, set the rank border (i.e., threshold; a list of 100 usually suffices, but the default is 1,000), and run the program (Figure 4E). Within a given sequence, idiomatic connections of triplets are highlighted, with core triplets in red and sub triplets in purple, and the ranks of idioms are shown immediately beside the sequence (Figure 4F). The well-known β-barrel structure of green fluorescent protein (GFP) is shown as an example. Idioms are found throughout the entire amino acid sequences, demonstrating the highly organized SCS usage of the GFP structure. We are now evaluating the performance of these idiom programs.

## Conclusions

Availability-based analyses are still in their infancy. More computational studies are necessary to construct a solid foundation for SCS usage bias in proteins. Direct applications to *in vivo* systems are just emerging. Furthermore, linguistic approaches, i.e., direct comparisons with languages using the availability-based concept as a tool, may enable a comprehensive understanding of protein language and may open up a new field of protein decoding and rational protein design.
